# Inhibition of VCP modulates NF-κB signaling pathway to suppress multiple myeloma cell proliferation and osteoclast differentiation

**DOI:** 10.18632/aging.204965

**Published:** 2023-08-21

**Authors:** Rongfang Wei, Yuhao Cao, Hongjie Wu, Xin Liu, Mingmei Jiang, Xian Luo, Zhendong Deng, Ze Wang, Mengying Ke, Yongqiang Zhu, Siqing Chen, Chunyan Gu, Ye Yang

**Affiliations:** 1 School of Medicine and Holistic Integrative Medicine, Nanjing University of Chinese Medicine, Nanjing, China; 2 College of Life Science, Nanjing Normal University, Nanjing, China; 3 Affiliated Hospital of Nanjing University of Chinese Medicine, Nanjing, China

**Keywords:** multiple myeloma, VCP, osteoclast differentiation, NF-κB, ubiquitination

## Abstract

Multiple myeloma (MM) is the second most common hematological malignancy, in which the dysfunction of the ubiquitin-proteasome pathway is associated with the pathogenesis. The valosin containing protein (VCP)/p97, a member of the AAA+ ATPase family, possesses multiple functions to regulate the protein quality control including ubiquitin-proteasome system and molecular chaperone. VCP is involved in the occurrence and development of various tumors while still elusive in MM. VCP inhibitors have gradually shown great potential for cancer treatment. This study aims to identify if VCP is a therapeutic target in MM and confirm the effect of a novel inhibitor of VCP (VCP20) on MM. We found that VCP was elevated in MM patients and correlated with shorter survival in clinical TT2 cohort. Silencing VCP using siRNA resulted in decreased MM cell proliferation via NF-κB signaling pathway. VCP20 evidently inhibited MM cell proliferation and osteoclast differentiation. Moreover, exosomes containing VCP derived from MM cells partially alleviated the inhibitory effect of VCP20 on cell proliferation and osteoclast differentiation. Mechanism study revealed that VCP20 inactivated the NF-κB signaling pathway by inhibiting ubiquitination degradation of IκBα. Furthermore, VCP20 suppressed MM cell proliferation, prolonged the survival of MM model mice and improved bone destruction *in vivo*. Collectively, our findings suggest that VCP is a novel target in MM progression. Targeting VCP with VCP20 suppresses malignancy progression of MM via inhibition of NF-κB signaling pathway.

## INTRODUCTION

ATPases associated with diverse cellular activities (AAA+ ATPase) are vital enzymes in all organisms, which are involved in various cellular processes, such as DNA replication, protein degradation, membrane fusion, signal transduction, and gene expression [1, 2]. Chemical inhibition on ATPase activity of the AAA+ ATPases impedes cancer progression, including multiple myeloma (MM) [3, 4]. The valosin containing protein (VCP, also known as p97 in mammals) is a member of the AAA+ ATPase family that is a multifunctional protein influencing protein metabolism and intracellular homeostasis to regulate cellular processes, such as cell cycle, DNA replication, virus replication, protein homeostasis, genome stability and repair, mitosis, and transcription [5–11].

It is known that VCP is elevated in many cancers and associated with poor prognosis, such as lymphoma [12], colorectal cancer [13], ovarian cancer [14] and prostate cancer [15]. Phospho-Ser^784^-VCP is required for DNA repair, checkpoint signaling, and cell survival in response to a broad range of genotoxins and correlates with poor outcomes in chemotherapy-treated breast cancer patients [16]. VCP regulates actin and cell motility via the Rho-ROCK dependent pathway in cancer development [17]. The interaction of VCP and ubiquitin regulatory X (UBX) cofactors mediates the binding of various E3 ligases to direct VCP for certain protein degradation processes [18]. VCP regulates the expression and function of the transcription factor c-Myc, and VCP-dependent degradation of ubiquitylated c-Myc on chromatin is important for promoting its function on gene expression [19]. In addition, VCP has been implicated in a variety of metabolic processes at the gene expression level in a diverse range of cancer cell lines and in patient-derived MM cells. The depletion of Glutamine leads to elevated expression of VCP, whereas VCP inhibition hampers the metabolic processes and intracellular amino acid turnover [20].

Recently, some studies have revealed that VCP is a potential target for cancer therapy including MM, and confirmed the significant biological effects of small molecule inhibitors against VCP [21–23]. Quinazoline-based VCP inhibitors induce G1 cell cycle arrest, decrease cap-dependent translation and mediate programmed cell death in ovarian cancer cells [14]. DBeQ, a selective and reversible inhibitor of VCP ATPase, arrests cell cycle at G1 phase and blocks the degradation of p21 and p27 in breast cancer cells [24]. In addition, DBeQ cooperates with bortezomib to induce MM cell apoptosis [22]. NMS-873 targeting a region in VCP spans D1 and D2 domains of adjacent polymers to activate the unfolded protein response (UPR), interferes with autophagy and induces cancer cell death [25]. Targeting VCP with CB-5083 (a potent inhibitor of VCP’s D2 ATPase domain) leads to an accumulation of poly-ubiquitinated proteins, retention of endoplasmic reticulum-associated degradation (ERAD) substrates, and generation of irresolvable proteotoxic stress, finally causes the activation of apoptotic arm of the UPR [26]. Additionally, CB-5083 promotes tumor cell killing following ionizing radiation both *in vitro* and *in vivo* [27]. However, due to the toxicity of CB-5083, two Phase I clinical trials of CB-5083 are discontinued. It is of great urgency to develop novel compounds to meet medical demand. Our team designed and synthesized a series of VCP inhibitors. Among these inhibitors, compound 35 (VCP20) exhibited significant *in vitro* activities and microsomal stabilities [28].

In the present study, we examined the inhibitory effect of VCP20 on MM models. Inhibition of VCP with VCP20 suppressed MM cell proliferation through inhibiting NF-κB signaling pathway. In addition, VCP20 inhibited osteoclast differentiation, however MM cell-derived exosomes attenuated its inhibitory effect probably due to the high expression of VCP in MM cell-derived exosomes. Furthermore, VCP20 prolonged the survival of MM model mice and improved their bone destruction. Taken together, we disclose that VCP is a potential therapeutic target for MM and VCP20 may be a promising agent for MM treatment.

## RESULTS

### Increased VCP expression correlates with poor survival in MM patients

**Figure 1:**
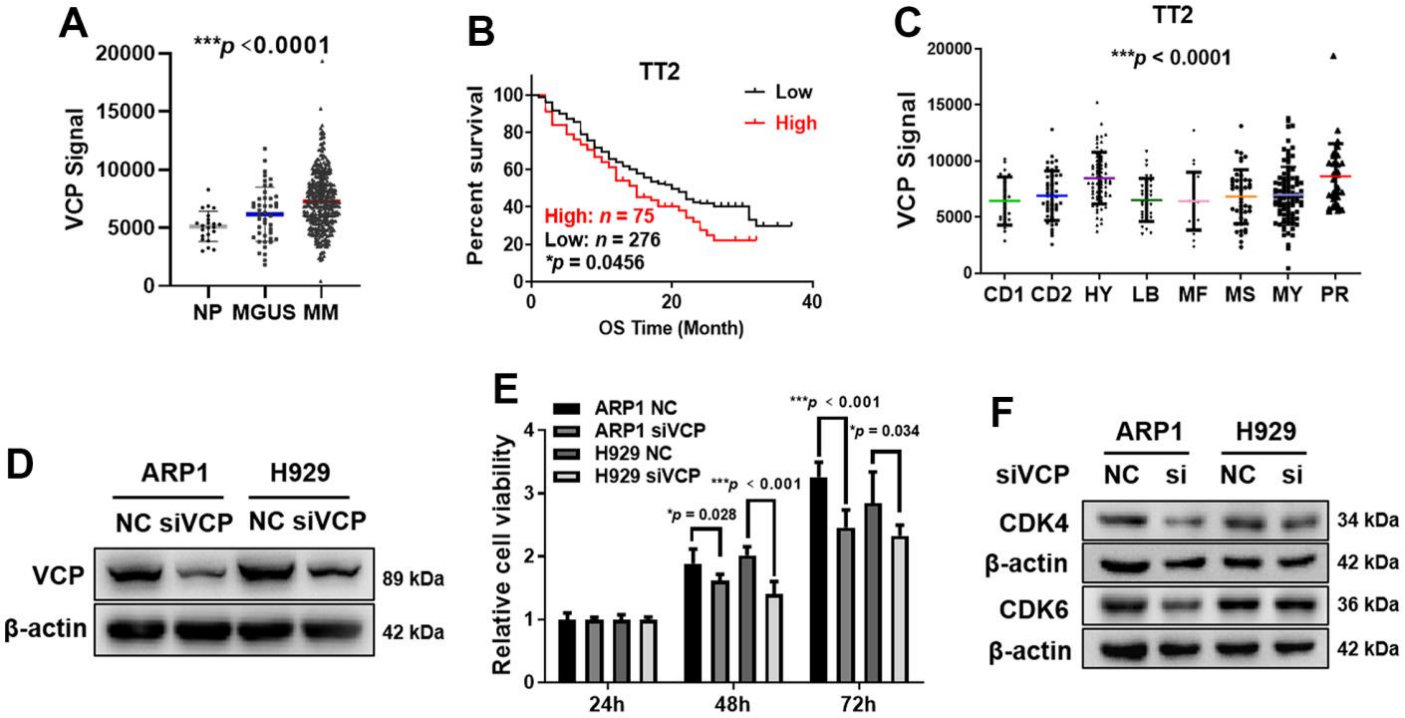
**Elevated VCP is associated with poor survival in MM patients and siVCP inhibits MM cell proliferation *in vitro*.** (**A**) VCP mRNA levels were significantly increased in MM samples (*n* = 351) compared with NP (*n* = 22) and MGUS (*n* = 44) samples. (**B**) Higher VCP level was associated with a shorter OS in TT2 cohort (*n* = 351, High: *n* = 75, Low: *n* = 276). (**C**) Box plot representing VCP expression in eight MM subgroups from TT2 cohort. (**D**) WB analysis of VCP expression in siVCP cells. (**E**) MTT assay examined the effect of siVCP on MM cell proliferation. (**F**) WB analysis showed that siVCP inhibited CDK4/6 expression in MM cells. The MM patient survival data were plotted by Kaplan-Meier curve, and the survival of patients with low and high expression of VCP was compared through a log-rank test. Data are presented as the mean ± SD; *p < 0.05; **p < 0.01; ***p < 0.001.

Initially, we examined VCP expression in normal plasma (NP, *n* = 22), monoclonal gammopathy of undetermined significance (MGUS, a pre-MM disease, *n* = 44) and MM samples (*n* = 351) from gene expression profiling (GEP) database. The levels of VCP were obviously increased in MM samples compared with NP and MGUS samples (***p < 0.0001) (Figure 1A). Notably, MM patients with high VCP expression suffered from poor clinical outcomes relative to low-VCP-expressing patients in TT2 cohort, which was presented as shorter response duration of overall survival (OS) (*n* = 351, *p = 0.0456) (Figure 1B). Furthermore, we tested the mean values of VCP levels in eight widely recognized subgroups, and found that elevated VCP was particularly prevalent in proliferation (PR) group, the worst subgroup in MM characterized by high proliferation (***p < 0.0001) (Figure 1C). Here, we assume that VCP may be a high-risk gene to promote MM progression.

### Downregulation of VCP inhibits MM cell viability *in vitro*

To examine whether VCP is an oncogene in MM, we interfered VCP expression by using small interfering RNA (siRNA) in ARP1 and H929 cells. WB analysis demonstrated successful knockdown of VCP in ARP1 and H929 cells at protein level (Figure 1D and Supplementary Figure 1A). Subsequently, we adopted MTT assay to explore the correlation between VCP and cell proliferation, which showed that MM cell proliferation was significantly decreased in siVCP cells compared with negative control (NC) after 72 h (***p < 0.001, *p = 0.034) (Figure 1E). As key regulators of cellular transition from G1 to S phase [29], CDK4/6 can promote cell cycle dysregulation and regulate cell proliferation in MM cells [30]. We measured CDK4/6 protein expression upon siVCP and found that interfering VCP decreased the expression of CDK4/6 (Figure 1F and Supplementary Figure 1A), suggesting that VCP participated in regulating MM cell cycle via altering CDK4/6 expression. Above results indicate that VCP may act as an oncogene in MM and targeting VCP leads to a decreased cell proliferation in MM.

### Compound VCP20 specifically targets VCP to impede MM cell proliferation and promote cell apoptosis *in vitro*

**Figure 2:**
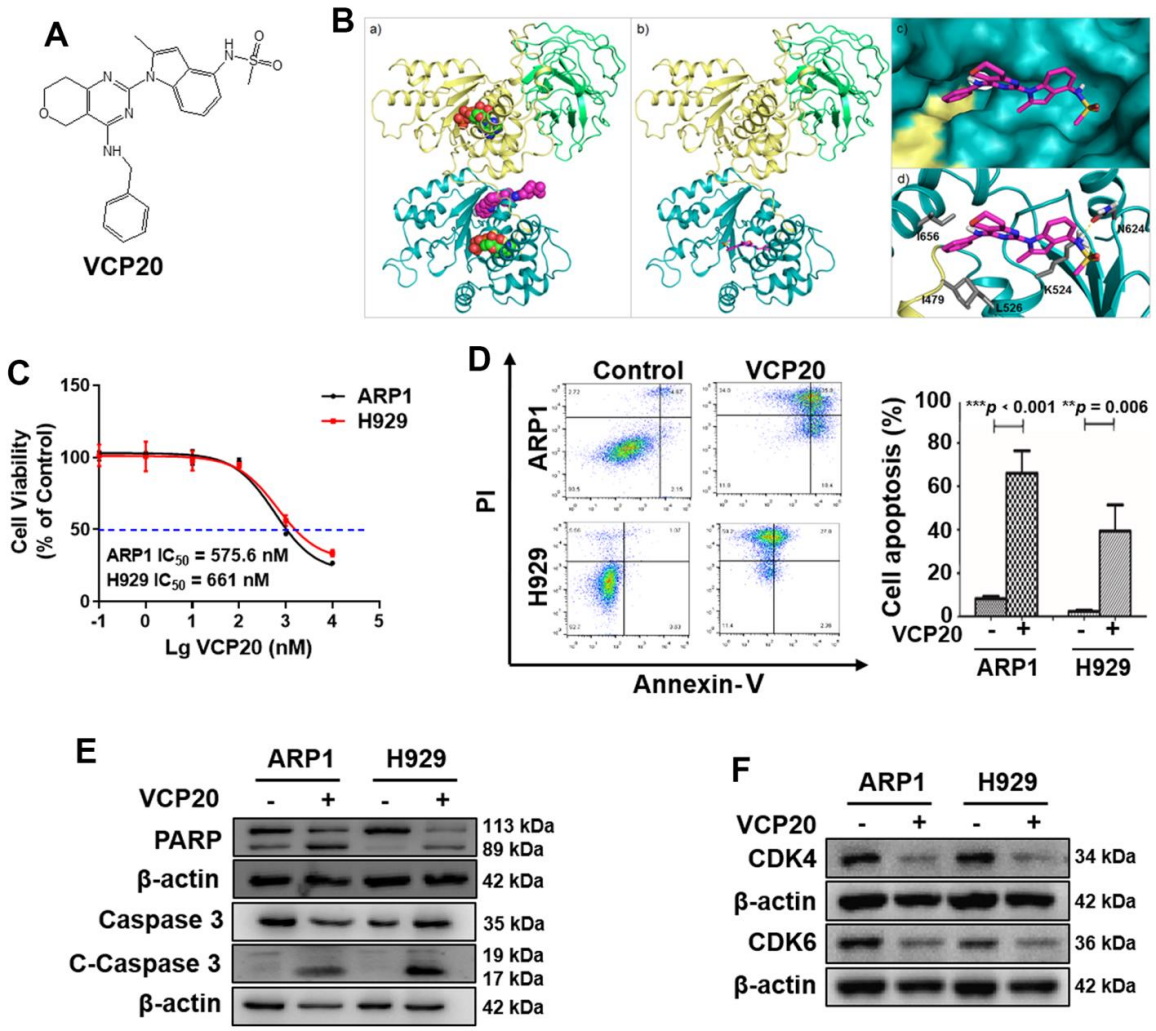
**VCP20 inhibits MM cell proliferation and induces cell apoptosis.** (**A**) The structure of VCP20. (**B**) Predicted binding modes of VCP20 targeting VCP. (**C**) Inhibition ratio of VCP20 in MM cells. (**D**) Flow cytometry analysis indicated that VCP20 induced MM cell apoptosis. (**E**) WB analysis showed that VCP20 increased the expressions of apoptotic protein. (**F**) WB analysis showed that VCP20 decreased the CDK4/6 expression. The concentration of VCP20 was as follow: ARP1, 575.6 nM; H929, 661 nM. Data are presented as the mean ± SD; **p < 0.01; ***p < 0.001.

CB-5083 was the first selective VCP inhibitor with the required pharmacological properties and showed promising preclinical activity, however it presented high toxicity in the clinical trials [26, 28]. It is certainly worthwhile to explore high-effective VCP inhibitors with low-toxicity. Therefore, our team synthesized a series of novel VCP inhibitors, including VCP20 (compound 35) with better VCP inhibitory activities (36.4 nM) (Figure 2A) [28]. Molecular docking of receptor-based 3D-QSAR models for VCP and VCP20 showed that VCP20 exclusively bound into VCP active pocket (Figure 2B), indicating that VCP20 specifically targeted VCP protein.

We performed MTT assay to evaluate the IC_50_ of VCP20 on ARP1 and H929 cells. The results showed that VCP20 suppressed MM cell proliferation evidently (ARP1, IC_50_ = 575.6 nM; H929, IC_50_ = 661 nM) (Figure 2C). Moreover, we tested the effect of VCP20 on the apoptosis level of MM cells. Flow cytometry assays demonstrated that VCP20 significantly induced MM cell apoptosis (***p < 0.001, **p = 0.006) (Figure 2D). We also detected the expressions of cleaved-PARP and cleaved-Caspase 3, and found that VCP20 induced the elevation of cleaved-PARP and cleaved-Caspase 3 in MM cells (Figure 2E and Supplementary Figure 1B). In addition, VCP20 decreased the expression of CDK4/6 (Figure 2F and Supplementary Figure 1B), which was consistent with the result in siVCP cells. These data indicate that VCP20 inhibits MM cell proliferation and accelerates cell apoptosis.

### VCP regulates MM cell proliferation via NF-κB signaling pathway

**Figure 3:**
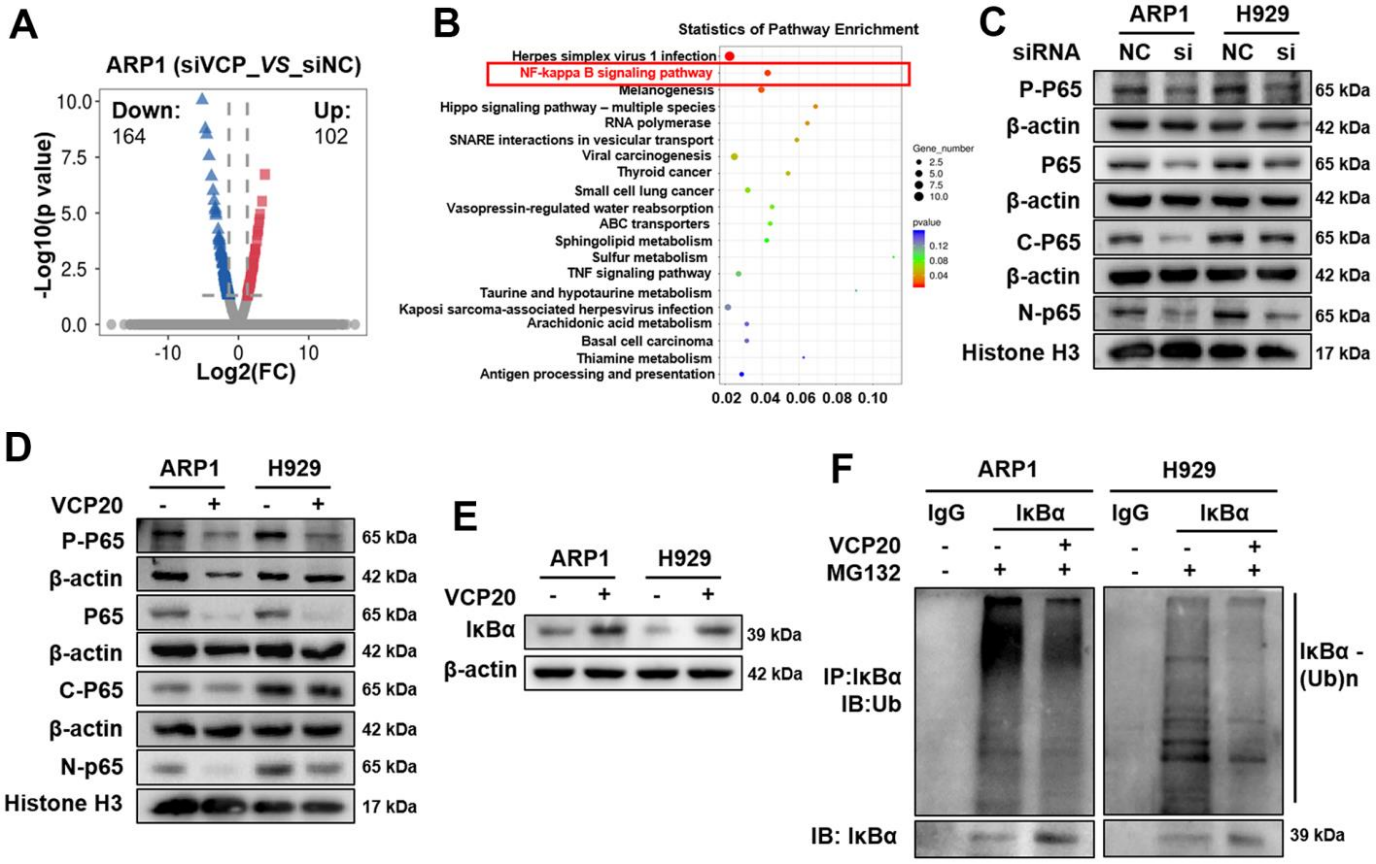
**VCP regulates MM cell proliferation via modulating NF-κB signaling pathway.** (**A**) Volcano plots showed the upregulated (red) and downregulated (blue) genes upon silencing VCP in MM cells. (**B**) KEGG pathway analysis of RNA-seq data indicated that VCP was associated with NF-κB signaling pathway. (**C**) WB analysis of P-P65 and P65 expressions in siVCP cells compared with NC cells. (**D**) WB analysis of P-P65 and P65 expressions in the cells treated with VCP20 compared with WT cells. (**E**) VCP20 increased IκBα expression. (**F**) The ubiquitination level of IKBα in MM cells with the treatment of VCP20 or not.

Since above findings have shed light on the vital role of VCP in MM progression, we followed to investigate the underlying mechanism. Through RNA-seq technology, we identified 164 downregulated genes and 102 upregulated genes between ARP1 siVCP and NC cells, and made the volcano plot of these differentially expressed genes. Volcano plot showed the upregulated (red) and downregulated (blue) genes upon silencing VCP in MM cells. (Figure 3A). KEGG pathway enrichment analysis for these differentially expressed genes demonstrated that the NF-kappa B (NF-κB) signaling was ranked in the top pathway (Figure 3B). It is known that NF-κB transcription factors play key roles in the pathogenesis of MM [31, 32]. We performed WB assay to examine the effect of VCP on the expressions of NF-κB P65 (P65) and phosphorylated-NF-κB P65 (P-P65) in MM cells. The expressions of P65 and P-P65 were observably decreased upon VCP inhibition. Furthermore, the expression of P65 in both of the cytoplasm and nucleus were significantly inhibited by siVCP (Figure 3C and Supplementary Figure 2A). Similar to the above results, the expressions of P65 and P-P65 were also downregulated in ARP1 and H929 cells with VCP20 treatment (Figure 3D and Supplementary Figure 2B). NF-κB activation is associated with the degradation of IκBα [33], and VCP interacts with IκBα to accelerate IκBα degradation and NF-κB activation [34]. Therefore, we detected the effect of VCP20 on IκBα protein expression. It was found that VCP20 enhanced IκBα protein expression remarkably (Figure 3E and Supplementary Figure 2B). Subsequently, we examined the effect of VCP20 on the level of IκBα ubiquitination. Intriguingly, ubiquitination of IκBα was decreased in both ARP1 and H929 cells after treated with VCP20 (Figure 3F). Taken together, these findings unveil that VCP20 inhibits NF-κB activation by decreasing the ubiquitination of IκBα to suppress MM cell proliferation.

### VCP20 inhibits osteoclast differentiation via inactivating NF-κB signaling pathway

**Figure 4:**
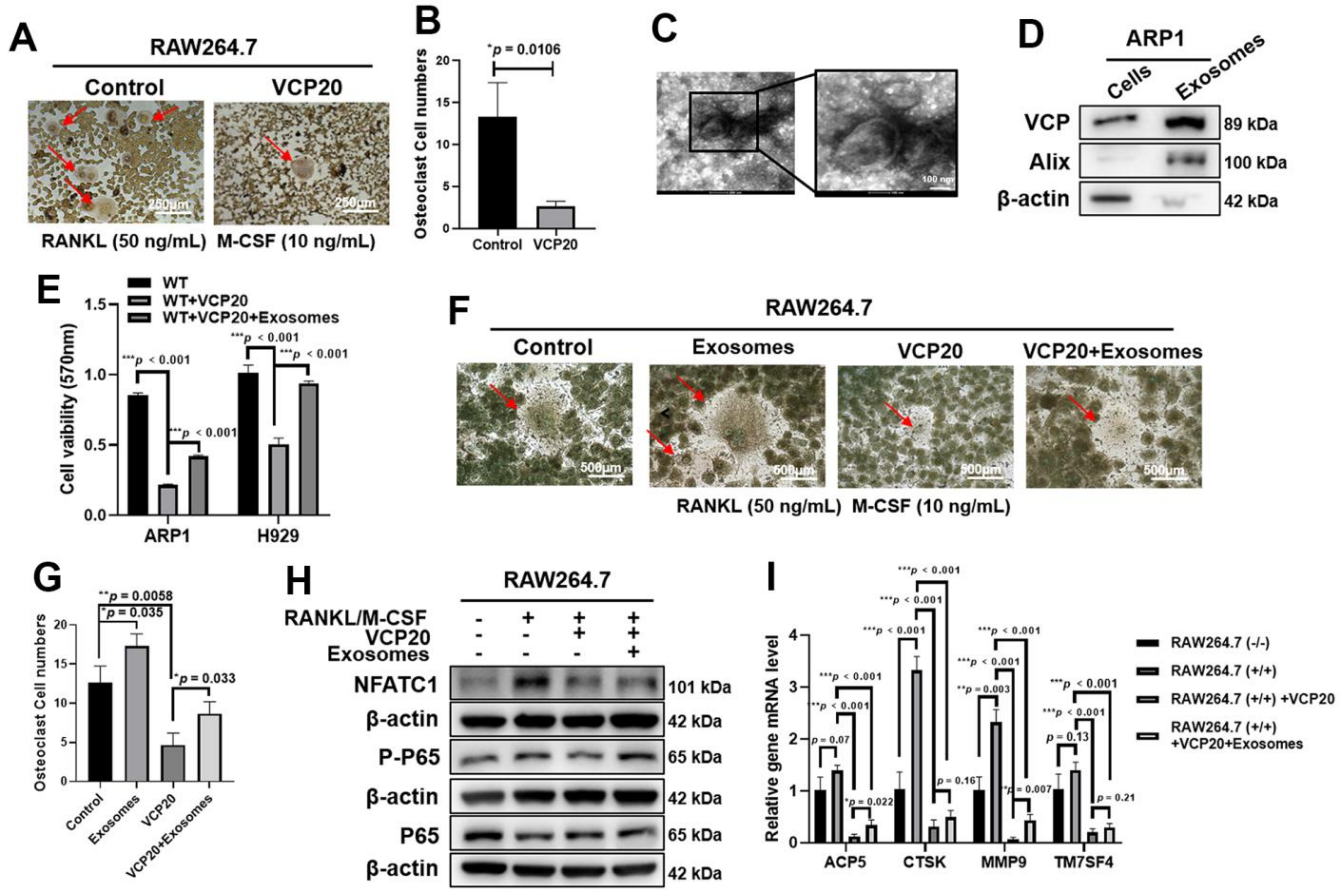
**VCP20 inhibits osteoclast differentiation via downregulating NF-κB P65.** (**A**) TRAP staining showed that VCP20 inhibited osteoclast differentiation in RAW 264.7 cells treated with RANKL and M-CSF. Scale bar: 250 μm. (**B**) Quantitative analysis of multinucleated osteoclasts. (**C**) The biological characteristics of exosomes were detected by transmission electron microscopy. Scale bar: 100 nm. (**D**) Detection of VCP, Alix and β-actin in ARP1 WT cells and exosomes from ARP1 WT cells by WB analysis. (**E**) Exosomes from ARP1 WT cells rescued cell proliferation inhibited by VCP20 in MM cells (1×10^6^ MM cells were treated with 20 μg exosomes or not for 48 h). (**F**) TRAP staining revealed that exosomes from ARP1 WT cells induced osteoclast differentiation. Scale bar: 250 μm. (**G**) Quantitative analysis of multinucleated osteoclasts. (**H**) WB assay confirmed that exosomes from ARP1 WT cells increased the expressions of P-P65 and NFATC1 that were downregulated by VCP20 in RAW264.7 cells. (**I**) RT-PCR assay showed that VCP20 decreased the levels of osteoclast differentiation related markers, and this effect was partially compensated by exosomes from ARP1 WT cells. VCP20: 100 nM; exosomes: 2 μg/mL each well. Data are presented as the mean ± SD; *p < 0.05; **p < 0.01; ***p < 0.001.

One of the characteristics of MM is bone disease caused by the imbalance between osteoclast and osteoblast activity [35]. NF-κB is a dimeric transcription factor complex that plays a vital role in osteoclastogenesis [36]. It has been confirmed that VCP is involved in MM progression via NF-κB pathway, hence we checked whether VCP20 would be associated with osteoclastogenesis. TRAP staining showed that VCP20 significantly inhibited the formation of osteoclast cells (*p = 0.0106) (Figure 4A, 4B). MM cell-derived exosomes can promote osteoclast differentiation and aggravate bone disease in MM [37, 38]. As VCP is associated with membrane fusion and considered as an exosome protein marker [39], we questioned whether VCP could exist in the exosomes derived from MM cells and promote osteoclast differentiation through exosomes. It was found that exosomes from ARP1 WT cells could be isolated by gradient ultracentrifugation, which were confirmed by using transmission electron microscopy (Figure 4C). The existence of VCP protein in exosomes derived from ARP1 WT cells was detected by WB method (Figure 4D). Furthermore, MTT assays showed that VCP20 inhibited MM cell proliferation significantly, meanwhile exosomes derived from ARP1 WT cells alleviated the inhibition effect of VCP20 on MM cell proliferation remarkably (***p < 0.001) (Figure 4E). We also detected the effects of exosomes extracted from ARP1 WT cells on osteoclastogenesis. TRAP activity results revealed that the exosomes significantly reversed the inhibition effect of VCP20 on the formation of osteoclast compared with Control cells (*p = 0.035, *p = 0.033, **p = 0.0058) (Figure 4F, 4G). Furthermore, the expressions of NFATC1 (an osteoclast marker [40]), P65 and P-P65 protein in RAW264.7 cells were examined by WB assay. WB results indicated that the expressions of NFATC1 and P-P65 were markedly decreased upon VCP20 treatment, in contrast exosomes derived from ARP1 WT cells partially increased the expressions of NFATC1 and P-P65 (Figure 4H and Supplementary Figure 3). In addition, RT-PCR analysis showed that the relative mRNA levels of osteoclast differentiation-related genes, such as *ACP5*, *CTSK*, *MMP9* and *TM7SF4*, were significantly decreased in VCP20 plus RANKL-induced RAW264.7 cells compared with RANKL-induced RAW264.7 cells, in contrast exosomes from ARP1 WT cells partially reversed the inhibitory effect (*p < 0.05, *p < 0.01, ***p < 0.001) (Figure 4I). Collectively, these findings suggest that VCP20 suppresses osteoclastogenesis via inhibiting NF-κB signaling pathway, and exosomes derived from MM cells partially abrogates the inhibition effect of VCP20 on osteoclastogenesis.

### VCP20 retards xenograft tumor formation, improves bone destruction and prolongs the survival of 5TMM3VT model mice *in vivo*

**Figure 5:**
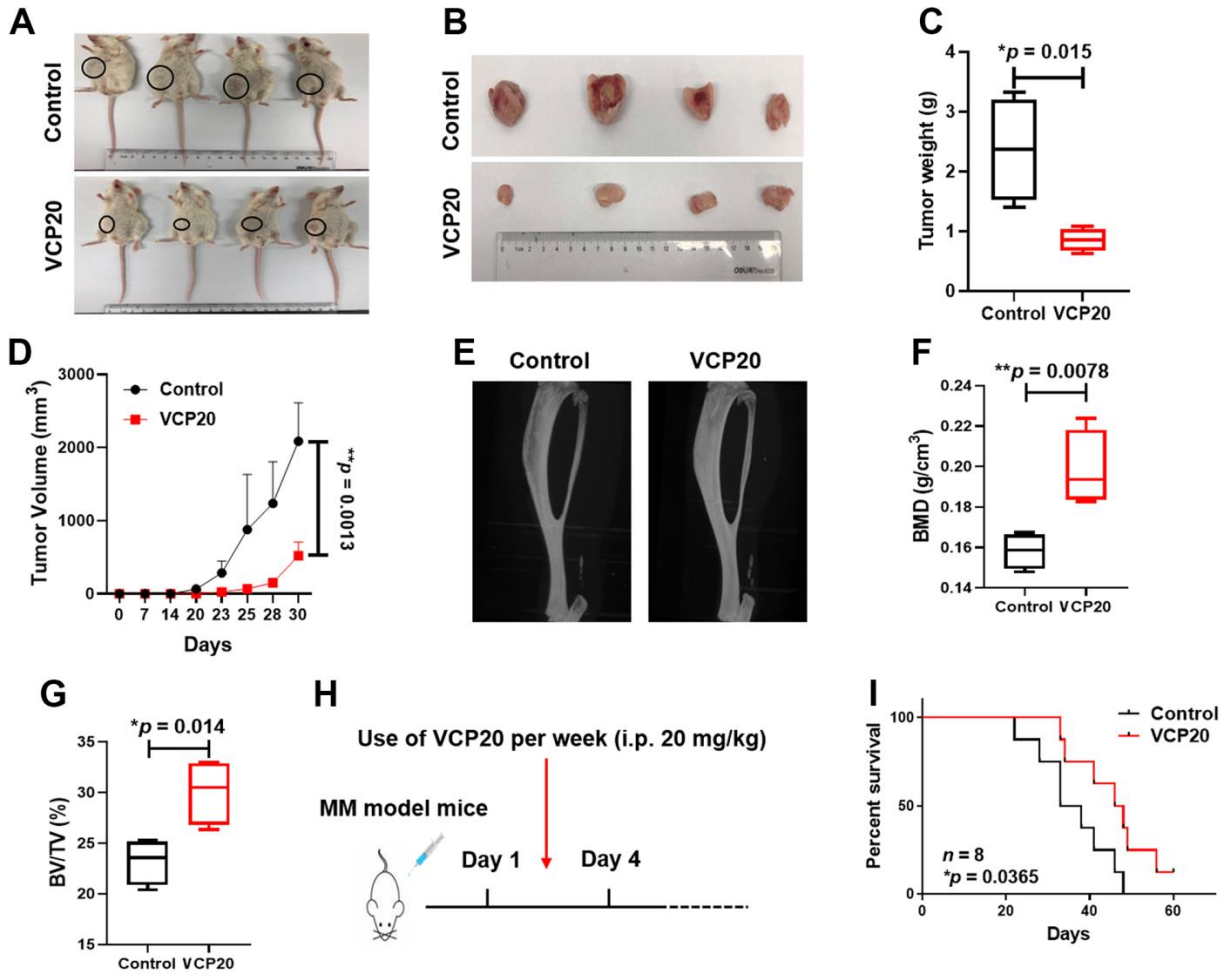
**VCP20 impedes MM cell proliferation and prolongs MM mice survival *in vivo*.** (**A**) Photographic images of xenograft mice at Day 31 (*n* = 4, VCP20: i.p. 20 mg/kg, twice a week). (**B**) Images of the harvested xenograft tumors. (**C**) Mean tumor weight of NOD/SCID mice. (**D**) Time course of tumor growth. *V* = 0.52 × larger diameter × (smaller diameter)^2^. (**E**) Representative micro-CT images of the bones. (**F**) BMD of 5TMM3VT mice in Control and VCP20 groups. (**G**) BV/TV of 5TMM3VT mice in Control and VCP20 groups. (**H**) VCP20 treatment started from the day after injection (VCP20: i.p. 20 mg/kg, twice a week) and continued until the mice were dead. (**I**) VCP20 extended the survival period of MM mice (*n* = 8). Data are presented as the mean ± SD; *p < 0.05; **p < 0.01.

As to further verify the role of VCP20 *in vivo*, we established cell-line derived xenograft model by injecting 1×10^6^ ARP1 WT cells subcutaneously to NOD/SCID mice (Control and VCP20; *n* = 4). After 31 days, the tumors in VCP20 group were much smaller than the tumors in Control group (Figure 5A, 5B). The mean weight of tumors in VCP20 group was significantly lower than that in Control group (*p = 0.015) (Figure 5C). Time course regression analyses of growth rate showed that the average volume of the tumors in VCP20 group were also significantly lower than that in Control group statistically (**p = 0.0013) (Figure 5D). Above data demonstrates that targeting VCP by VCP20 retards the development of MM *in vivo,* and VCP20 may act as a promising compound for MM treatment.

In addition, we employed 5TMM3VT mouse model to evaluate the effect of VCP20 on osteoclastogenesis *in vivo*. After one month of modeling, the 5TMM3VT model mice presented paralysis of the hind limbs. Micro-CT analysis indicated that the bone destruction was milder in VCP20 mice than Control mice (Figure 5E). The differences between bone mineral density (BMD) and bone volume fraction (BV/TV) in two groups were deeply analyzed. The mean BMD was 0.158 g/cm^3^ and BV/TV was 23.2% in control mice, by contrast the mean BMD was 0.198 g/cm^3^ and BV/TV was 30.06% in VCP20 mice (**p = 0.0078, *p = 0.014) (Figure 5F, 5G), suggesting that VCP20 significantly improved bone destruction in MM mice. We further confirmed the therapeutic effect of VCP20 on the survival of MM mice. It was found that VCP20 significantly prolonged the survival of MM mice relative to the untreated mice (*p = 0.0365) (Figure 5H, 5I). All the data above demonstrate that targeting VCP by VCP20 improves bone marrow environment and prolongs the survival of MM mice *in vivo*.

## DISCUSSION

MM is a plasma cell malignancy characterized by complex heterogeneous cytogenetic abnormalities [41]. Although novel targeted drugs have shown great prospects in overcoming conventional drug resistance and improving patient prognosis, MM remains incurable. Therefore, it is an emergency to seek novel targets and related inhibitors to improve MM treatment. To date, elevated VCP expression has been identified in many cancer types and associated with patient outcomes, which is correlated with aggressiveness and drug resistance [18, 42, 43]. VCP promotes cancer cell proliferation or invasion and metastasis in lymphoma [12], colorectal cancer [13], prostate cancer [15], lung cancer [34], breast cancer [16, 44] and pancreatic cancer [45]. In present study, we investigated the function of VCP in MM to confirm if VCP was representing a potential therapeutic target, and identified that a specific VCP inhibitor (VCP20) targeted VCP to inhibit MM cell proliferation and osteoclast differentiation.

We clarified that VCP mRNA level was elevated in MM, which was associated with poor survival of patients with MM. Schweitzer K et al. have demonstrated that VCP promotes Cullin-RING-ubiquitin-ligase/proteasome-dependent degradation of IκBα [46]. Silencing VCP leads to depressed IκBα degradation and NF-κB activation [34]. Our data confirmed that inhibition of VCP significantly inactivated NF-κB signaling pathway to impede MM cell proliferation. It is a promising strategy to develop small-molecule inhibitors of VCP for cancer treatment. The reported inhibitors of VCP include CB-5083 [45], CB-5339 [47], NMS-873 [48], PPA [42], DBeQ [49], OSSL_325096 [21], FQ393 [50] and so on. CB-5083 entered two phase I clinical trials, but the trials were halted for the toxicities caused by off-target effects [51, 52]. VCP20 (compound of 35) is a novel pyrimidine structure VCP inhibitor designed from CB-5083, which shows excellent enzymatic inhibitory activity [28]. In this study, VCP20 was employed to treat MM *in vitro* and *in vivo*. VCP20 significantly inhibited MM cell proliferation via inhibiting the ubiquitination degradation of IκBα to inactivate NF-κB signaling pathway.

Like most malignancies, MM cells alter its microenvironment to promote tumor progression that confers resistance to conventional therapy [53]. Paget's disease of bone (PDB) is a common skeletal disorder characterized by abnormal focal bone remodeling [54], in which VCP is a susceptibility gene in PDB pathogenesis [55]. We assumed that VCP might participate in the regulation of bone marrow microenvironment. We examined whether VCP20 could affect osteoclast differentiation. Intriguingly, VCP20 evidently inhibited osteoclast differentiation to improve bone lesion, suggesting that targeting VCP might be an impactful way to ameliorate bone marrow microenvironment. Exosomes secreted by stromal cells and tumor cells are critical regulators of intercellular communication in tumor microenvironment, which are particularly suitable for the transport and intracellular delivery of proteins and nucleic acids [56, 57]. Tumor-derived exosomes remodel the bone marrow environment, inhibit anti-leukemia immunity, mediate drug resistance and interfere with immunotherapies in hematological malignancies [58]. MM cell-derived exosomes promote osteoclast differentiation to support MM cell proliferation [37]. Of note, we identified VCP existing in MM cell-derived exosomes, which indicated that VCP could be released to bone marrow microenvironment to take effect.

Alberto Bosque et al. have reported that targeting VCP may be a new selective strategy to prevent tumoral exosome secretion in cancer treatment [59]. Our further study revealed that MM cell-derived exosomes significantly reversed the inhibitory effect of VCP20 on MM cell proliferation and osteoclast differentiation, indicating that VCP released by exosomes promoted MM progression and VCP20 could alleviate the cell proliferation promotion caused by VCP secretion. Moreover, mechanism study indicated that VCP20 inhibited NF-κB signaling pathway and suppressed NFATC1 expression, leading to restraining osteoclastogenesis. To further extend our research, we employed 5TMM3VT mouse model to validate the therapeutic effect of VCP20 *in vivo*. We observed that VCP20 significantly prolonged the survival of MM mice and improved the bone destruction. In the present study, we revealed that VCP20 effectively inhibited MM cell proliferation and bone lesion formation through targeting VCP in the bone marrow microenvironment.

**Figure 6:**
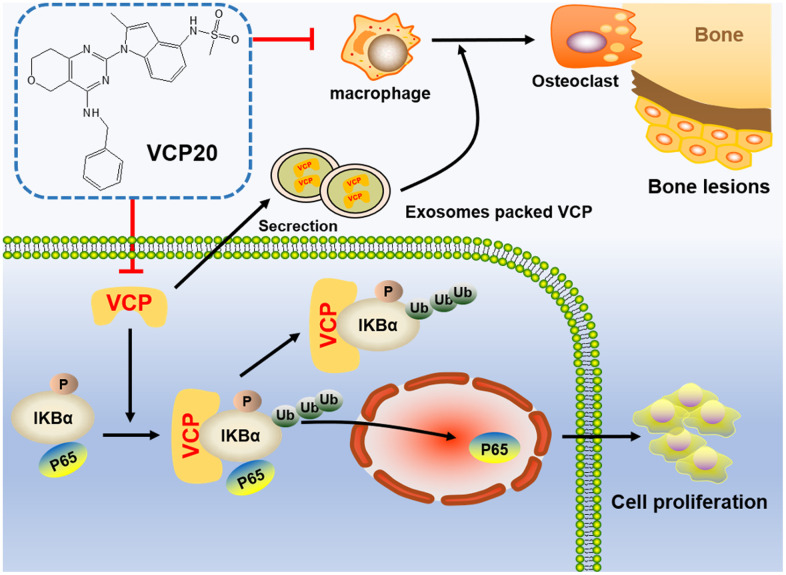
Schematic depiction illustrates that VCP20 targeting VCP is a promising agent for inhibiting cellular proliferation and improving bone marrow microenvironment in MM.

In conclusion, we demonstrate for the first time that VCP20 targeting VCP is a promising agent for inhibiting MM cell proliferation and improving bone marrow microenvironment in MM. Figure 6 illustrates the carcinogenesis role of VCP in MM and inhibition of VCP impedes MM progression via NF-κB signaling pathway. Targeting VCP with VCP20 may thus be a novel strategy in the treatment of MM.

## MATERIALS AND METHODS

### Gene expression profiling

The Gene expression profiling (GEP) cohorts were collected from GEO database. Total therapy 2 (TT2) cohort was collected from GSE2658.

### Antibodies and reagents

The following antibodies were used, VCP (10736-1-AP, Proteintech, Wuhan, China), P65 (8242S, Cell Signaling Technology, Danvers, MA, USA), P-P65 (3033S, Cell Signaling Technology), PARP (9542S, Cell Signaling Technology), Cleaved Caspase-3 (9661S, Cell Signaling Technology), CDK4 (11026-1-AP, Proteintech), CDK6 (14052-1-AP, Proteintech), Histone H3 (17168-1-AP, Proteintech), CD63 (216130, Abcam, Cambridge, UK), IκBα (9242S, Cell Signaling Technology), Ubiquitin (43124S, Cell Signaling Technology), β-actin (60008-1-Ig, Proteintech). Secondary antibodies included goat anti-Rabbit IgG (H+L) HRP (FMS-Rb01, Fcmacs, Nanjing, China) or mouse (S0002, Affinity, Changzhou, China). MTT was purchased from Solarbio (M8180, Beijing, China). Propidium iodide (PI) was purchased from Beyotime (ST511, Shanghai, China), Annexin-V-FITC was purchased from Biolegend (640906, San Diego, CA, USA). Compound VCP20 was obtained from Professor Yongqiang Zhu (Nanjing Normal University).

### Cell lines and cell culture

Human MM cell lines, ARP1 and H929, were cultured in RPMI 1640 medium (Biological Industries, Kibbutz Beit Haemek, Israel) and RAW264.7 were cultured in DMEM medium (Biological Industries) containing 10% fetal bovine serum (Invigentech; Irvine, CA, USA), penicillin and streptomycin solution (NCM, Suzhou, China). All cells were cultured in humidified 5% CO_2_ incubators at 37° C.

### Transient transfection

Small interfering RNA (siRNA) was transfected into MM cells by using electro transmitter (BTX, Holliston, MA, USA). The process was performed as described previously [60]. The specific siRNAs were synthesized by Shanghai Genepharma. The sequences of siRNAs were as following: negative control (NC, sense 5'-UUCUCCGAACGUGUCACGUTT-3' and anti-sense 5'-ACGUGACACGUUCGGAGAATT-3'); VCP (sense 5'- CCAACAGACCCAACAGCAUTT-3' and anti-sense 5'- AUGCUGUUGGGUCUGUUGGTT-3').

### Cell proliferation and viability assay

MM cells (5×10^3^) were seeded in each well of 96-well plate. The relative cell viability was calculated as the ratio of absorbance at a certain time relative to the mean value of 24-hour absorbance.

As to detect the effect of VCP20 on MM cell proliferation, cells were treated with different concentrations of VCP20 for 48 h to calculate the IC_50_ values in MM cells. Cell viability was evaluated by MTT assay according to the manufacturer’s instructions. The absorbance was finally determined at 570 nm using the microplate reader (Thermo Fisher, Manassas, VA, USA). The results from vehicle-treated cells were considered as 100% viability.

### Transcriptomic RNA-sequencing (RNA-seq)

ARP1 NC and ARP1 siVCP cells were used for RNA-seq analyses. The procedure of RNA-seq was performed as previously described [61]. All data analysis and processing were performed by Lc-Bio Technologies (Hangzhou) Co., Ltd. (Hangzhou, China). We have uploaded the data of RNA-seq in GEO database (GSE210920, https://www.ncbi.nlm.nih.gov/geo/query/acc.cgi?acc=GSE210920).

### *In vivo* ubiquitylation assay

MM cells were incubated with 20 μM MG132 for 12 h before collection, and lysed in IP lysis buffer, followed by Co-IP and Western blotting (WB) analyses. Ubiquitylation assay was performed according to the protocol of the Pierce™ Direct Magnetic IP/Co-IP kit (88828, Thermo Fisher, Waltham, MA, USA). Briefly, the cell lysate was subjected to immunoprecipitation with IκBα antibody, and immunoprecipitation was subsequently separated by SDS-PAGE and immunoblotted with a Ubiquitin antibody to detect the ubiquitination level of IκBα. WB was performed as described previously [61].

### Molecular docking based alignment

The crystal structure of VCP was retrieved from Protein Data Bank (https://www.rcsb.org/), and was prepared by the Molecular Operating Environment (MOE) software (Chemical Computing Group, Inc.: Montreal, Canada). The binding mode between VCP20 and VCP was manually built and refined by MOE.

### Flow cytometry analysis of cell apoptosis

Cell apoptosis was analyzed by flow cytometry as reported in the literatures [62]. Flow cytometry equipped with Guava easyCyte System (Merck Millipore, Darmstadt, Germany) was applied to detect cell apoptosis.

### Isolation and application of exosomes

Exosomes were purified from MM cell-derived conditioned medium. The conditioned medium was collected after 48 h and centrifuged at 300×g for 10 min at 4° C, followed by 1,000×g for 10 min and 10,000×g for 30 min at 4° C. The supernatant was ultracentrifuged at 100,000×g for 70 min at 4° C. The exosomes were washed with PBS followed by a second ultracentrifugation at 100,000×g for 70 min at 4° C and then resuspended in PBS.

### Tartrate-resistant acid phosphatase (TRAP) activity staining

RAW264.7 cells (1.5×10^3^) were seeded in 24-well plate with recombinant murine sRANKL (315-11, Peprotech, Cranbury, NJ, USA) and M-CSF (315-02, Peprotech) on Day 2. The cells were treated with VCP20 (100 nM) or exosomes (2 μg/mL each well). After 6 days, the cells were stained for TRAP activity.

### Real-time PCR

TRIeasy Total RNA Extraction Reagent (19201ES60, Yeasen, Shanghai, China) was used to extract total RNA. Complementary DNA was synthesized by using reverse transcription kit (11123ES10, Yeasen). Real-time quantitative PCR was performed with SYBR Green master Mix (11198ES03, Yeasen). The Sequences of primers were as follows, GAPDH: Forward Sequence CATCACTGCCACCCAGAAGACTG and Reverse Sequence ATGCCAGTGAGCTTCCCGTTCAG; ACP5: Forward Sequence GCGACCATTGTTAGCCACATACG and Reverse Sequence CGTTGATGTCGCACAGAGGGAT; CTSK: Forward Sequence AGCAGAACGGAGGCATTGACTC and Reverse Sequence CCCTCTGCATTTAGCTGCCTTTG; MMP9: Forward Sequence GCTGACTACGATAAGGACGGCA and Reverse Sequence TAGTGGTGCAGGCAGAGTAGGA; TM7SF4: Forward Sequence TTTGCCGCTGTGGACTATCTGC and Reverse Sequence GCAGAATCATGGACGACTCCTTG.

### MM xenografts

ARP1 WT cells (2×10^6^) were injected subcutaneously into abdominal of 6~8 weeks old NOD/SCID mice and divided into two groups randomly (Control and VCP20, *n* = 4 per group). On Day 3 after cell injection, intraperitoneal administration of VCP20 (20 mg/kg) was performed twice weekly in VCP20 group. In all cases, tumor diameter was measured with calipers 2~3 times weekly. When the tumor diameter reached 15 mm, the mice were sacrificed. All animal work was performed in accordance with government-published recommendations for the Care and Use of laboratory animals and guidelines of Institutional Ethics Review Boards of Nanjing University of Chinese Medicine (Ethics Registration No. 201905A003).

### 5TMM3VT MM mouse model

5TMM3VT cells (1×10^6^) were injected through the tail vein into C57BL/KaLwRij mice from Harlan Laboratories (Indianapolis, IN, USA). The mice were divided into two groups (Control and VCP20). On Day 3 after cell injection, intraperitoneal administration of VCP20 (20 mg/kg) was performed twice weekly in VCP20 group. The mice of tibia model were sacrificed by spinal dislocation. The bone mineral density (BMD) and bone volume faction (bone volume/total volume [BV/TV]) of the tibia were analyzed by micro-computed tomography (micro-CT) (SkyScan 1176, Bruker microCT, Germany) to assess osteolysis. The death time of the mice was recorded to generate the survival curve (*n* = 8 per group).

### Statistical analysis

All values were expressed as means ± SD. Two-tailed Student’s t-test and One-way analysis of variance (ANOVA) (≥ three groups) were used to determine significance between experimental groups. The Kaplan-Meier method was used to evaluate the correlation of VCP expression with MM patient survival. In all cases, significance was defined as *p* < 0.05.

## Supplementary Material

Supplementary Figures
