# Supplementary material for: Inhibition of VCP modulates NF-κB signaling pathway to suppress multiple myeloma cell proliferation and osteoclast differentiation
Source: Aging (Albany NY). 2023 Aug 21;15(16):8220–36. doi: 10.18632/aging.204965 (PMC10497005; doi:10.18632/aging.204965)
Supplement: Supplementary Figures [file aging-15-204965-s001.pdf]

SUPPLEMENTARY FIGURES

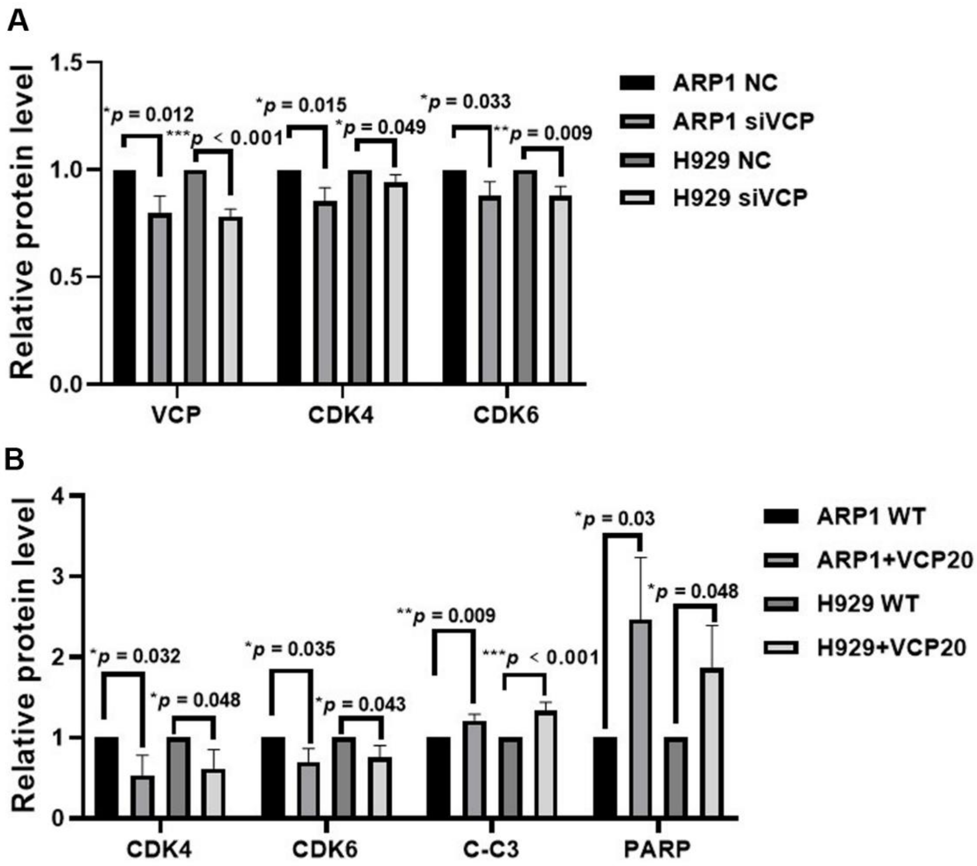

Supplementary Figure 1. Quantitative analysis of WB results. (**A**) Quantitative analysis of VCP, CDK4, and CDK6 expressions in siVCP and NC cells. (**B**) Quantitative analysis of CDK4, CDK6 C-C3 and PARP expressions in cells treated with VCP20. The data are expressed as mean ± SD. ($^{*}p < 0.05$; ** $p < 0.01$; $^{***}p < 0.001$). Related to Figures 1D, 1F, 2E, 2F in the manuscript.

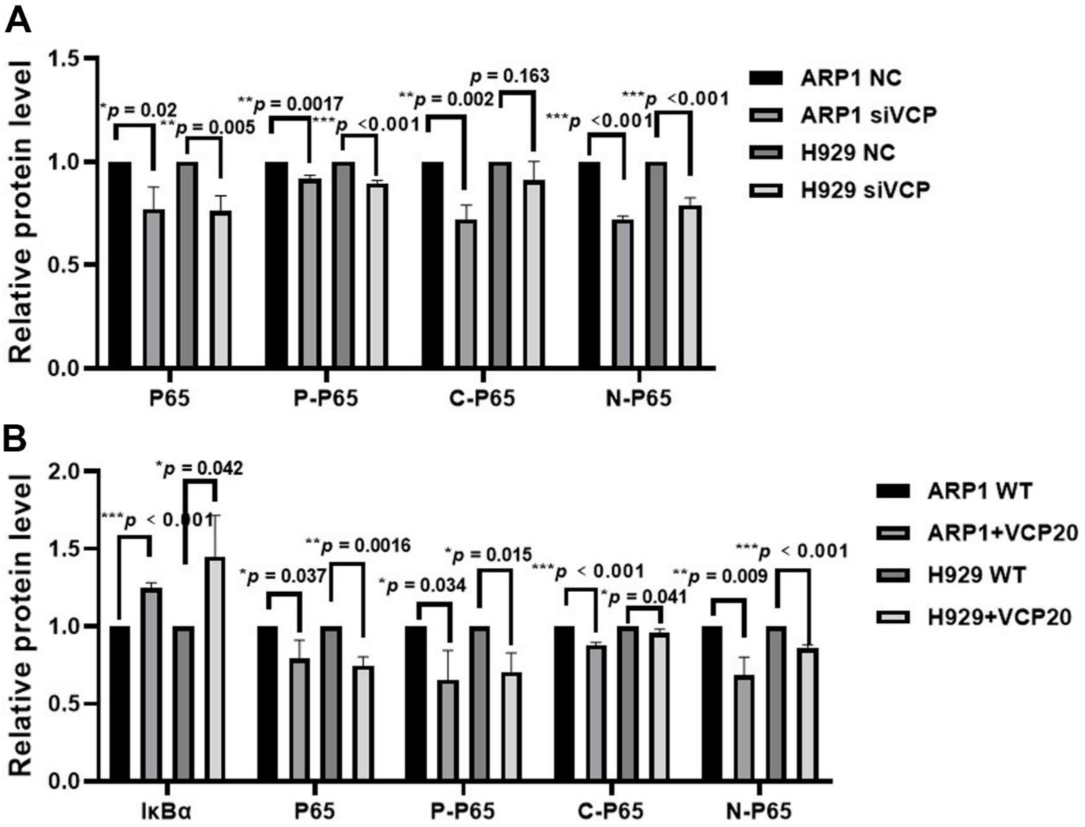

Supplementary Figure 2. Quantitative analysis of WB results. (**A**) Quantitative analysis of P65, P-P65, C-P65 and N-P65 expressions in siVCP and NC cells. (**B**) Quantitative analysis of IκBα, P65, P-P65, C-P65 and N-P65 expressions in cells treated with VCP20. The data are expressed as mean ± SD. ($^{*}p < 0.05$; $^{**}p < 0.01$; $^{***}p < 0.001$). Related to Figure 3C–3E in the manuscript.

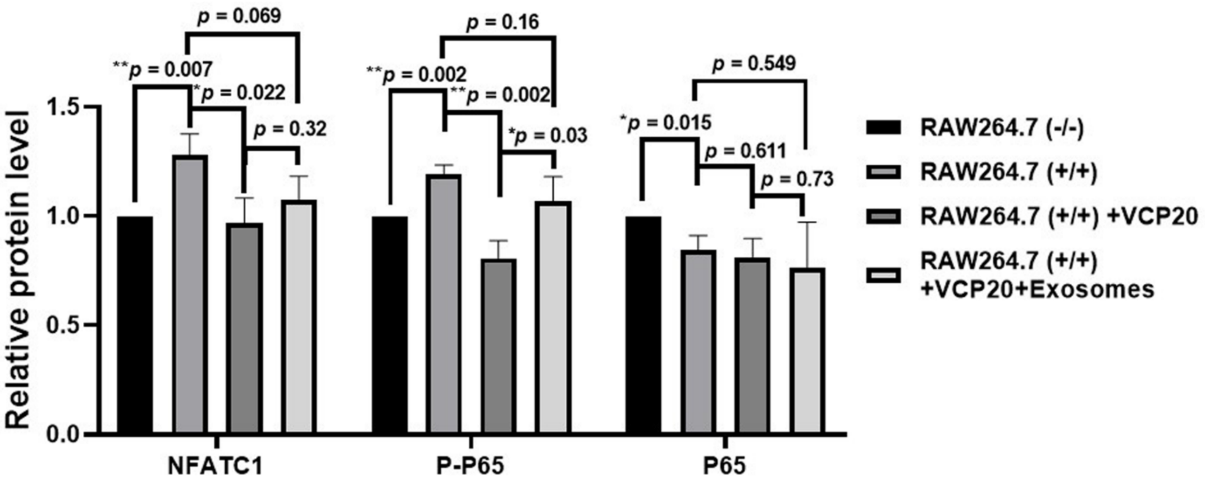

Supplementary Figure 3. Quantitative analysis of NFATC1, P-P65 and P65 expressions in RAW264.7 cells treated with RANKL/M-CSF, VCP20, Exosomes or not. The data are expressed as mean ± SD. ($^{*}p < 0.05$; $^{**}p < 0.01$). Related to Figure 4H in the manuscript.
